# Antitumor Assessment of Liposomal Beta-Carotene with Tamoxifen Against Breast Carcinoma Cell Line: An In Vitro Study

**DOI:** 10.3390/biom15040486

**Published:** 2025-03-26

**Authors:** Marim H. Elsayed, Medhat W. Shafaa, Mohga S. Abdalla, Manal F. El-Khadragy, Ahmed E. Abdel Moneim, Shimaa S. Ramadan

**Affiliations:** 1 Molecular Biotechnology Sector, Chemistry Department, Faculty of Science, Helwan University, Cairo 11795, Egypt; marimhesham544@gmail.com; 2 Medical Biophysics Division, Physics Department, Faculty of Science, Helwan University, Cairo 11795, Egypt; shafaa@science.helwan.edu.eg; 3 Biochemistry Sector, Chemistry Department, Faculty of Science, Helwan University, Cairo 11795, Egypt; mohga.shafiek@science.helwan.edu.eg (M.S.A.); shimaa.shawki@yahoo.com (S.S.R.); 4 Department of Biology, College of Science, Princess Nourah bint Abdulrahman University, P.O. Box 84428, Riyadh 11671, Saudi Arabia; mfelkhadragy@pnu.edu.sa; 5 Zoology and Entomology Department, Faculty of Science, Helwan University, Cairo 11795, Egypt

**Keywords:** beta-carotene, tamoxifen, liposomes, DSC, FTIR, apoptosis

## Abstract

The present study was designed to characterize the interactions between lecithin liposomes, a model membrane, and either β-carotene or tamoxifen. In addition, the cytotoxicity of liposomal beta-carotene with tamoxifen was screened in vitro in human breast cancer cell lines MCF-7 and MDA-MB-231 in addition to the normal WI38 cell line. All liposomes were nearly spherical and evenly distributed and had fewer aggregates for encapsulated and empty vesicles. Measurements using dynamic light scattering verified that each sample was monodisperse. When tamoxifen is incorporated into liposomal membranes, the zeta potential values tend to decrease. In the test for cytotoxicity using MCF-7 treated cells, the liposomal β-carotene IC_50_ value was at least 0.45 μg/mL, whereas the IC_50_ of free β-carotene treated cells was 7.8 μg/mL. For MCF-7 treated cells treated with free tamoxifen, the IC_50_ was 9.92 μg/mL, but for its liposomal form, it was 20.88 μg/mL. According to the cytotoxicity test using MDA-MB-231 treated cells, the IC_50_ values for free tamoxifen, free β-carotene, liposomal β-carotene, liposomal tamoxifen, and liposomal tamoxifen β-carotene were 15.5 μg/mL, 38.1 μg/mL, 12.1 μg/mL, 21.2 μg/mL, and 11.4 μg/mL, respectively. This investigation demonstrated that free β-carotene has a more potent cytotoxic impact than tamoxifen. The findings showed that each comet assay variable for the liposomal β-carotene was significantly (*p* < 0.05) elevated in comparison with tamoxifen and control values. Analysis using flow cytometry revealed that the MCF-7 cells displayed a greater degree of cell apoptosis than the control cells following a 48 h exposure to liposomal β-carotene. Based on available data, a novel treatment plan that includes liposomal β-carotene may boost antitumor activity toward the MCF-7 cancer cell line. The current findings demonstrated that preparations of natural products might be a good substitute for pharmaceutical interventions in the treatment of breast cancer.

## 1. Introduction

Cancer and other noncommunicable diseases (NCDs) are recognized as a global health issue for which there is no common treatment [1]. The term “cancer” refers to a group of illnesses marked by uncontrollably spreading and abnormally growing cells, which, if unchecked, often end in death. It is currently the second most frequent reason for demise, with the mere condition that has higher mortality rates than heart disease. Surgical excision, chemotherapy, radiotherapy, immunotherapy, and targeted treatment are among the available treatment options [2].

Breast cancer has the second-highest death rate among all cancers. By 2050, according to World Health Organization (WHO) estimates, there will be a remarkable 27 million additional cases of breast cancer globally, and the disease is expected to claim 17.5 million people’s lives. [3]. Numerous risk factors, including race, ethnicity, genetic characteristics, family history of cancer, and modifiable exposures, including alcohol use, physical inactivity, exogenous hormones, and specific female reproductive variables, can cause breast cancer [1]. Breast cancer is curable if caught early, especially if it has only spread to the axillary lymph nodes or is still confined to the breast regions. Improvements in multimodal treatment have raised the likelihood of recovery for 70–80% of patients. In contrast, breast cancer at its advanced (metastatic) stage is thought to be incurable with current treatment options [4].

Considerable advancements have been achieved in the management of cancer thanks to the utilization of nanocarriers, especially lipid-based ones. Lipid formulations in several forms have been developed, including lipid nanoparticles and nanostructured lipid carriers. Compared to other drug delivery methods such as polymeric nanoparticles, these lipid-based models are less hazardous because of their benefits in biocompatibility and biodegradability [5].

In particular, liposomes are synthetic spherical vesicles with a hollow core surrounded by a lipid bilayer. They can be directed toward tumor locations and filled with chemotherapy medications. Drug delivery systems have embraced liposomes because of their efficacy, biocompatibility, non-immunogenicity, improved drug solubility, and capacity to encapsulate a variety of agents. By avoiding healthy tissues and possibly lowering the dosage needed to have cytotoxic effects in tumor cells, liposomal delivery of anticancer medications improves their effectiveness [6].

Triglycerides, glycolipids, and different phospholipids, including phosphatidylcholine, phosphatidylethanolamine, and phosphatidylinositol, are all complicated mixtures that make up lecithin. Nonetheless, pure phosphatidylcholine, a crucial phospholipid mostly present in the phosphate portion of egg yolk, is referred to as lecithin in the biochemistry community. Known to be a safe surfactant, lecithin is readily accepted by living things. It is a vital part of cell membranes and is fully metabolized by the body. For the purpose of creating liposomal structures, lecithin is the perfect option due to its accessibility and safety profile [7].

Numerous clinical trials have looked into the use of carrier systems to increase the specificity of therapeutic medication delivery. In this context, liposomes have been investigated as doxorubicin and cyclophosphamide carriers, two anticancer medications [8,9]. Research on mice has demonstrated that anticancer medications enclosed in liposomes are significantly less harmful than those that are not. Furthermore, when liposomes are injected intravenously, they primarily target organs with high reticuloendothelial cell concentrations. Therefore, liposomal delivery of antitumor drugs will enhance part of their efficacy by steering the drug away from healthy tissue or minimizing the quantity required to have a cytotoxic impact on tumor cells [8].

As adaptable delivery systems for different drug encapsulation, liposomes present a viable way to reduce the toxicity problems associated with chemotherapeutic medications [10,11]. By removing the pharmacokinetic interactions between PTX and both DXR and its metabolite, doxorubicin, Franco et al. [12] discovered that a 1:10 co-encapsulation ratio of PTX and DOX in liposomes improved the cardiac toxicity profile in mice with the 4T1 breast tumor. Furthermore, the liposomes functionalized with wheat germ agglutinin (WGA) and co-loaded with tetrandrine (TET) and DNR were described by Liu et al. [13] to increase cellular uptake through receptor-mediated endocytosis. Inhibiting the expression of P-glycoprotein (P-gp), TET, which was loaded and distributed into the lipid bilayers, made it possible to overcome chemoresistance. The functionalized liposomes dramatically enhanced the production of pro-apoptotic proteins (Bax and Bak), activated caspase 8, 9, and 3 apoptosis pathways, and efficiently accumulated in cancer cells (MCF-7 and MCF-7/ADR), according to in vitro experiments. Additionally, liposomal drug delivery may be able to stop breast cancer from spreading. Both the vasculogenic mimicry channels (VMC) and the epithelial-to-mesenchymal transition (EMT) have been implicated in the metastasis and chemoresistance of breast cancer in the past ten years [14]. Indeed, there is growing evidence that multidrug resistance (MDR) can be overcome by using liposomal drug delivery systems [15].

Plant-derived carotenoids are a class of lipophilic pigments with a polyisoprenoid structure. According to epidemiological studies, higher circulating levels of dietary carotenoids, including β-carotene, α-carotene, lutein/zeaxanthin, and lycopene, have been associated with a decreased incidence of breast cancer [16]. Owing to the high concentration of β-carotene found in these plant-based meals, it has been thoroughly studied as a possible cancer-prevention tool. Several studies have highlighted the anti-carcinogenic properties of β-carotene, which is present in our daily diet together with other antioxidant molecules [17,18]. Moreover, earlier research has shed light on the various biological characteristics of β-carotene, such as its anti-inflammatory, antioxidant, and anticancer actions. Its potential as a useful element in cancer prevention and treatment strategies has been further cemented by the demonstration of its aptitude for growth inhibition and cytotoxicity across a variety of cancer cell lines and animal models [19].

The purpose of using natural supplements for cancer is to lessen drug resistance. They work synergistically with anticancer medications to reduce drug concentrations, which in turn lessens the negative side effects of cancer treatment and prevents harm to normal, healthy cells [20].

One common non-steroidal antiestrogen medication used to treat and prevent breast cancer is tamoxifen. Tamoxifen and other antiestrogens inhibit the growth of tumors by competing with endogenous estrogen-receptor binding sites, which is the mechanism behind their antitumor action [21].

The relationship between phospholipids and tamoxifen, β-carotene, or a combination of the two has not been studied before; this includes the behavior of phospholipids in the thermotropic phase, changes in acyl chain conformations, and the identification of distinctive PO_2_^−^ bands in the phospholipid’s polar heads. Furthermore, there is still unknown information regarding the anti-proliferative properties of tamoxifen, β-carotene, or both when applied to the cell line for breast cancer MCF-7, whether in their free forms or encapsulated in nanoliposomes.

Using lecithin as a model lipid membrane, this study sought to determine how β-carotene or tamoxifen affected the physical characteristics of lecithin. To precisely evaluate changes in the lipid bilayer structure, a number of analytical methods were used, such as zeta potential measurement, polydispersity analysis, transmission electron microscopy, dynamic light scattering, differential scanning calorimetry, and Fourier transform infrared spectroscopy. Additionally, the cytotoxic efficacy of liposome-encapsulated β-carotene or free tamoxifen was assessed. The study’s objective was to clarify any possible impact these substances might have on the in vitro survival of the breast cancer cell lines.

## 2. Materials and Methods

### 2.1. Chemicals

**Figure 1:**
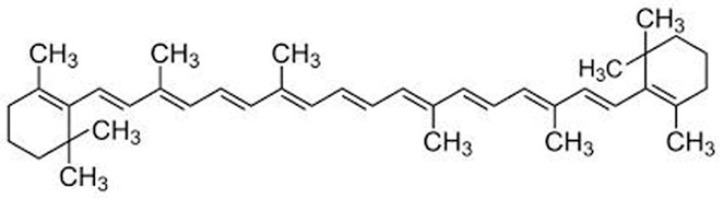
The chemical structure of β-carotene.

**Figure 2:**
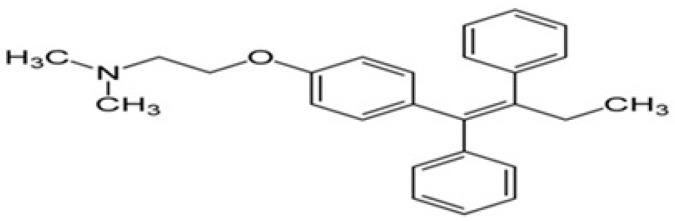
The chemical structure of tamoxifen.

**Figure 3:**
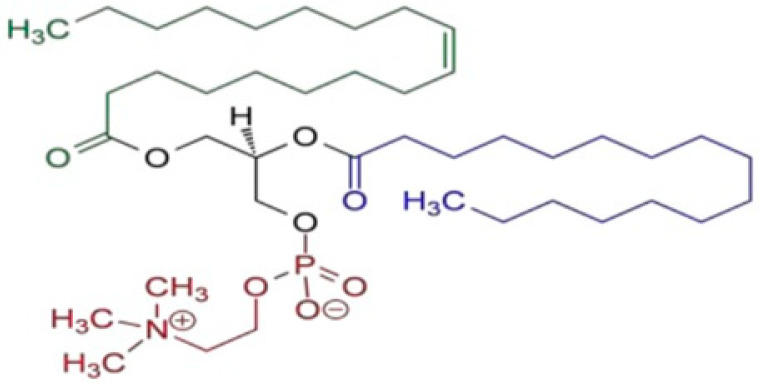
Schematic chemical structure of L-α-phosphatidyl choline (soy lecithin).

β-carotene was carefully extracted and refined from natural sources, and its purity was thoroughly confirmed by means of thin-layer chromatography (TLC) in addition to high-performance liquid chromatography (HPLC). The β-carotene structure, which has a molecular weight of 536.9, was verified by spectral data (Figure 1). A 371.515 molecular weight tamoxifen was acquired from Pharmachemie (Haarlem, The Netherlands). Figure 2 depicts the tamoxifen molecule’s structure. DaeJung Chemicals (Seohaean-ro, Gyeonggi-do, Republic of Korea) provided the completely pure 99.9% ethanol. As shown in Figure 3, powdered L-α-phosphatidylcholine (also known as soy lecithin) with a purity ≥97% and molecular weight of 760 was acquired from Carl Roth (Karlsruhe, Germany). The source of the phosphate buffer saline was CDH in New Delhi, India. MCF-7, a human breast cancer cell line, was cultivated serially at the “VACSERA Vaccination Centre, Cairo, Egypt” at −180 °C (liquid nitrogen) in accordance with “The American Form Culture Array” methods. Sigma Chemical Co., St. Louis, MO, USA, provided the following: dimethylsulphoxide (DMSO), DMEM medium, sodium bicarbonate, Penicillin/Streptomycin, acetic acid, Trypsin, fetal bovine serum (FBS), trichloroacetic acid (TCA), and sulphorhodamine-B (SRB). Furthermore, 100% isopropanol and SRB (0.4%) dissolved in acetic acid (1%) were obtained for testing. Every solution was made using ultra-pure distilled water, and all reagents and solvents were HPLC grade.

### 2.2. Preparation of Liposomes

Soy lecithin: Tamoxifen was utilized in a 7:2 molar ratio to create neutral MLVs by Bangham’s technique (thin film hydration) [22]. In a 100 mL flask, 100 mg of soy lecithin and 14 mg of tamoxifen were added, either with or without 20 mg of β-carotene at a 2:7 molar ratio. After adding 25 milliliters of ethanol (EtOH), the flask was shaken to ensure that all of the ingredients had dissolved. To create a consistent lipid thin layer on the inside flask wall, the organic solvent was steadily evaporated by the use of a rotary evaporator operating under vacuum in a warm (55 °C) bath of water. To create multilamellar vesicles (MLVs), hydration of the lipid film was performed with phosphate buffer saline (pH 7.4) in a water bath at 55 °C for fifteen minutes. The flask was shaken at 55 °C for 1 h. After that, a nitrogen stream was used to cleanse the flask, and it was promptly stopped. Using solely the soy lecithin mass aliquots that were initially employed in production, empty liposomes, which serve as the control, were created using the previously described method.

### 2.3. Measurement of Encapsulation Efficiency

The effectiveness of tamoxifen’s encapsulation into soy lecithin liposomes was obtained by removing untrapped tamoxifen using centrifugation at 6000 rpm for 20 min. The amount of unloaded tamoxifen in the supernatant was determined by using a UV-Visible spectrophotometer (Jasco V-630, Hachioji, Tokyo, Japan) at a wavelength of 235 nm. The wavelength was changed to 235 nm (tamoxifen resonance absorption peak). The supernatant absorption for every sample was contrasted to the standard curve for the absorption of various tamoxifen concentrations.

Tan et al. [23] stated that extraction was used to determine the beta-carotene encapsulation efficiency. To determine the amount of free β-carotene in suspension, 1 mL of beta-carotene loaded on liposomes was vortexed with ethyl acetate (3 mL) at room temperature for three minutes. The supernatant was separated from the centrifuged sample by centrifuging the combined sample for five minutes at 2000 rpm. The above-mentioned process was repeated twice. Lastly, ethyl acetate was added to a tube containing the combined supernatant. The free quantity of carotenoid in beta-carotene was quantified spectrophotometrically at 452 nm using ethyl acetate (blank). Each test was conducted in triplets.

### 2.4. Entrapment Efficiency Percentage

Entrapment efficiency (EE%) was estimated by using the following equation [24]:

(1) $$EE\%=\frac{Total\;drug\;imput\left(\mathrm{mg}\right)-Drug\;in\;supernatant(\mathrm{mg})}{Total\;drug\;input(\mathrm{mg})}\times 100$$

### 2.5. Dynamic Light Scattering and Zeta Potential

Using a particle sizing method for dynamic light scattering in Tris-buffer (pH = 7.4) at 25 °C, the mean size of particles, size distribution, and zeta potential of the empty liposomes and liposomes coated with either tamoxifen, β-carotene, or tamoxifen together with β-carotene, were determined. All results are shown as mean ± standard deviation, and the experiment was run in triplicate.

### 2.6. DSC Measurements

Differential scanning calorimetry (Shimadzu, Kyoto, Japan) model DSC-50 calibrated using indium was utilized to examine the thermal characteristics of lyophilized specimens of empty liposomes and liposomes conjugated with either tamoxifen, β-carotene, or tamoxifen combined with β-carotene. Five-milligram sealed samples are analyzed in conventional aluminum pans. The thermogram of each sample scans at a rate of 3 °C per minute over a range of temperatures from 25 to 200 °C.

### 2.7. FTIR Spectroscopy

The FTIR spectra of lyophilized empty liposomes and liposomes coated with β-carotene, tamoxifen, or tamoxifen plus β-carotene were obtained using a JASCO FT/IR-4100 spectrometer (Jasco, Tokyo, Japan). Within the 400–4000 cm^−1^ range, the scanning was performed at room temperature at a 2 mm/s speed.

### 2.8. Cytotoxicity In Vitro Using the MTT Assay

The human breast cancer cell lines MCF-7 and MDA-MB-231, in addition to the normal WI38 cell line, were washed with PBS and trypsinized, followed by resuspension in the culture medium of RPMI-1640; then, cells were counted by the trypan blue exclusion technique using a hemocytometer chamber [25]. For studies concerning chemosensitivity, the culturing of cells was performed in a 96-multi-well plate at a density of 10^4^ cells per well for 24 h prior to using the chemotherapy to enable the cells to attach to the plate wall. The medium was extracted from each well after 24 h of incubation and swapped with a new cell culture medium that includes the compounds investigated at various levels (0–1000 µg/mL) using a serial dilution (two-fold) procedure. The potential cytotoxicity of empty liposomes, free tamoxifen, free β-carotene, and liposomes coupled with either tamoxifen, β-carotene, or tamoxifen paired with β-carotene was tested separately. For each drug, the experiment was performed for every dose in triplicate wells.

Following that, cell monolayers were incubated for 48 h at 37 °C with 5% CO_2_ and various chemotherapeutic agent concentrations. After eliminating the drug-containing medium, the cells were twice washed with PBS. A total of 50 μL of yellow methyl thiazolyl tetrazolium (MTT; 0.5 mg/mL) salt was added right away to each well, and the plates were then incubated for 4 h at 37 °C. After extracting the medium, DMSO (50 μL) was added to dissolve the formazan crystals.

In order to estimate the percentage of viable cells, the absorption value of the resultant color was identified at 490 nm using an ELISA reader (Boster Immunoleader, Pleasanton, CA, USA). The treated wells’ true absorbance divided by the controls and declared as a percentage was used to calculate cell survival. To determine the half-maximal inhibitory concentration (IC_50_) or the drug amount that suppresses cell growth by 50%, the correlation between the viability of cells and medication concentration was drawn. This allowed for the creation of a survival curve for the breast cancer cell lines versus a given drug.

### 2.9. Single-Cell Gel Electrophoresis (Comet Assay)

The comet assay was carried out according to Singh et al. [26] using modifications suggested by Blasiak et al. [27]. Layers of cell microgels were created. A pre-cleaned microscope charged slides were coated with 100 μL of agarose (0.7%) of normal melting point (m.p.) and gently covered with a coverslip to create the first layer of gel. When the agarose reached a solid state (4 °C), the coverslip was taken off. At 37 °C, low m.p. agarose (0.5%) was prepared in 100 mmol/LPBS. A total of 100 µL of a mixture containing mononuclear cells and low m.p. agarose was added to the first gel layer. Subsequently, the slides were placed under the coverslip and allowed to solidify at 4 °C. When the second layer developed, the coverslips were taken out of the cell microgels. After applying the last layer of low m.p. agarose and letting them solidify for ten minutes, the coverslips were removed.

The slides were covered with 100 milliliters of fresh lysis buffer (pH = 10) for 1 h at 4 °C. The buffer contained 2.5 mol/L NaCl, 1% sodium hydroxide, 100 mmol/L EDTA, 1% Triton X-100, 10 mmol/L Tris, and 10% DMSO. After draining and being treated for 30 min at 4 °C with a DNA unwinding solution (1 mmol/L EDTA, 300 mmol/L NaOH, pH 13), microgel slides were immediately put into a horizontal gel electrophoresis room that was filled with the solution. Gels were run continuously at 4 °C with a 300 mA current for 30 min. After electrophoresis, the microgels were neutralized for ten minutes at pH 7.5 using a 0.4 M Trisma base. Slides were stained with 20 μL of 10 µg/mL ethidium bromide.

The slides were viewed at 400 magnification using a fluorescent microscope (IX70; Olympus, Tokyo, Japan) with a 549 nm excitation filter and a barrier filter of 590 nm, connected to a video camera (Olympus, Japan). With a brilliantly glowing head and a tail to one side caused by breaks of DNA strands that were pulled away during electrophoresis, each cell had the appearance of a comet. Counting the cells that were damaged out of one hundred cells on each of the slides allowed us to determine the percentage of damage.

### 2.10. Apoptosis Detection Using Flow Cytometry

The MCF-7 cancer cell line was treated for 48 h at a density of 1 × 10^6^ cells/25 cm^2^ culture flask using the IC_50_ concentration of the three tested samples—free β-carotene, blank liposomes, and liposomes doped with β-carotene—as determined by the MTT assay. The cells were then trypsinized and centrifuged for 10 min at 2000 rpm, with the supernatant removed. The residual cells were then cleaned with a cold PBS buffer. The supernatant was disposed of after centrifuging for 5 min at 500× *g* at 4 °C. The cell pellets were then resuspended in 100 μL of binding buffer that enclosed 5 μL propidium iodide (PI) and 1 μL Annexin V-FITC. This was done at room temperature and in the dark for fifteen minutes. Lastly, 400 μL of binding buffer was put in prior to the BD FACSCalibur flow cytometer analysis. Apoptotic profiles were used to express the apoptosis analysis results.

### 2.11. Analysis of Statistics

The mean standard deviation was used to display the results for continuous variables. The unpaired *t*-test was used to evaluate continuous variable differences between two groups. Two-tailed *p*-values with 95% confidence intervals were used. By contrasting treated samples with untreated control, statistical analysis was carried out. Prism software version 8.0 (GraphPad, San Diego, CA, USA) was used for all statistical tests.

## 3. Results and Discussion

### 3.1. Characterization of Tamoxifen Combined with β-Carotene into Liposomes

**Figure 4:**
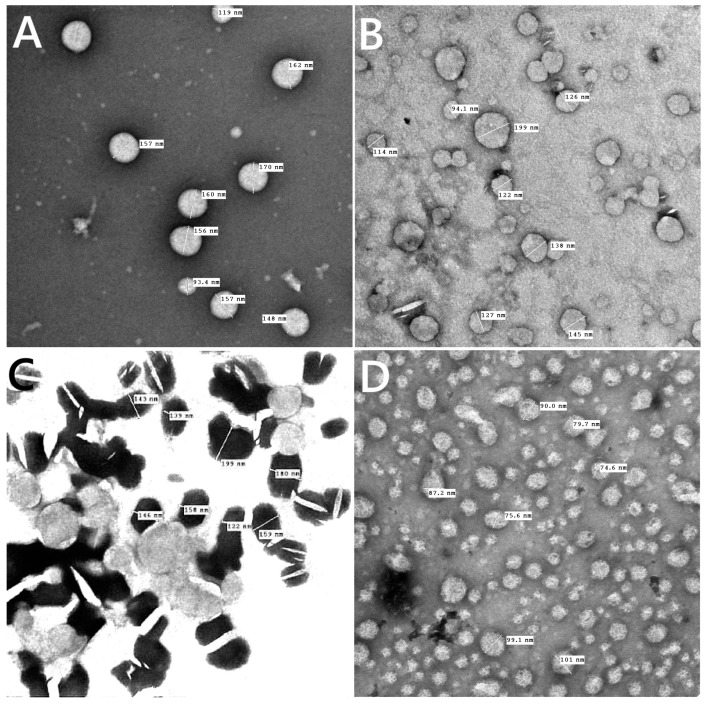
TEM images for blank liposomes (**A**), tamoxifen-loaded liposomes (**B**), β-carotene-loaded liposomes (**C**), and tamoxifen combined with β-carotene into liposomes (**D**).

For every prepared liposomal suspension, the percent of entrapment efficiency was found to be greater than 90% when the drug was combined with lipid powder and then dissolved in ethanol. TEM images showed that, as shown in Figure 4A–D, all of the liposomes made in this work had an almost spherical shape, were evenly distributed, and had less aggregates for β-carotene vesicles that were empty or encapsulated. TEM findings showed that β-carotene was physically associated with causing liposomes’ surface membrane packing to be disrupted (Figure 4C). The presence of β-carotene in liposomes may induce stronger interactions via hydrogen bonding with the liposome lipid bilayer. The presence of β-carotene in liposomes enlarged the distance between adjacent bilayers, leading to liposomes that were bigger than the controls. Stronger drug interactions with the liposome lipid bilayer via hydrogen bonds may be the cause of the boost in particle size. These results could be explained by the nonpolar beta-carotene’s random distribution throughout the lipid bilayer, which has no preferred orientation [28]. This gives the alkyl hydrocarbon chains more mobility, which could account for the size increase. The possibility of inserting β-carotene in the hydrophobic part of the bilayer is supported, and these results are consistent with the data from the FTIR and DSC analyses.

Particle size is determined using a method called dynamic light scattering (DLS). The homogeneity of colloidal suspension particles is effectively taken into account by the polydispersity index (PDI). A sample with a very wide size distribution and potentially unstable stability for the DLS technique is indicated by values greater than 0.7 [29].

**Figure 5:**
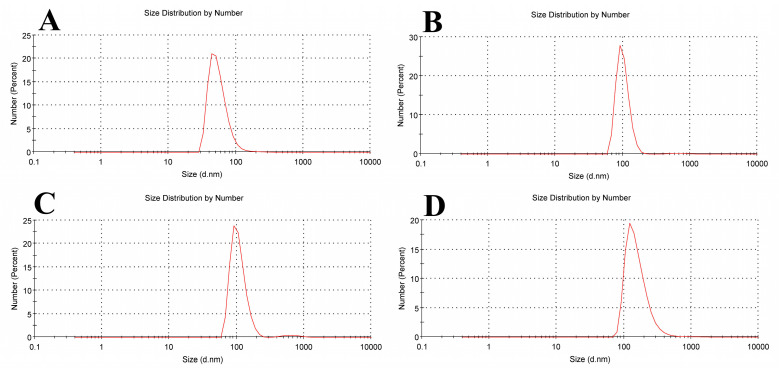
Liposome size distribution measured by dynamic light scattering (DLS) for (**A**) empty soy lecithin liposomal sample, (**B**) tamoxifen-encapsulated liposomes, (**C**) β-carotene-encapsulated liposomes, and (**D**) tamoxifen combined with beta-carotene into liposomes.

A pure soy lecithin liposomal sample’s size distribution is depicted in Figure 5A. It was concentrated at a mean diameter of 43.82 ± 21.86 nm with a 0.477 PDI. Figure 5B shows that when tamoxifen is encapsulated into soy lecithin, the mean size diameter of pure soy lecithin was increased to 91.28 ± 22.13 nm with 0.546 PDI, and this is most likely explained by a greater electrostatic repulsive force present between the tamoxifen NH^3+^ group and the soy lecithin N(CH_3_)^3+^ group inside the liposome lipid bilayer. Tamoxifen is primarily thought of as a lipophilic drug and may become trapped in the bilayer hydrophobic core. These findings show that the addition of tamoxifen to liposomes increased the distance between neighboring bilayers, which led to the formation of larger liposomes than those in the control ones. When soy lecithin liposomes were used to encapsulate β-carotene, the mean vesicle sizes were dramatically increased (91.28 ± 27.26 nm with 0.674 PDI) (Figure 5C). According to these findings, the beta-carotene molecule appears to be submerged in a lipid bilayer that is randomly distributed and lacks any desired orientation. This raises the motion of the alkyl chains, which could account for the size increase. After tamoxifen bound with β-carotene at a molar ratio (1:1) was added to soy lecithin liposomes, Figure 5D demonstrates a significant rise in the mean size of empty soy lecithin liposomes to 122.4 ± 83.37 nm with 0.408 PDI. According to the results above, tamoxifen increases the liposomal core size, and β-carotene may increase the size of the lipid bilayer. These outcomes closely match the data found in the DSC and FTIR investigations.

The potential stability of the colloidal system is indicated by the magnitude of zeta potential. A more stable dispersion of colloids will result from an increase in particle repulsion caused by an increase in zeta potential. Particles in suspension that exhibit strong positive or negative zeta potentials tend to repel one another, making it unlikely for the particles to assemble [30].

Empty liposomes displayed negative zeta potential (−23.8 ± 12.4 mV), in line with other research findings [31,32,33]. As a result of tamoxifen being integrated into the liposomal membranes, tamoxifen-loaded liposomes had a lower negative zeta potential (−17.6 ± 11.7 mV) than blank liposomes. The addition of β-carotene to liposomal membranes seems to stabilize the density of negative charge (−23.5 ± 14.9 mV), stabilizing the zeta potential.

**Figure 6:**
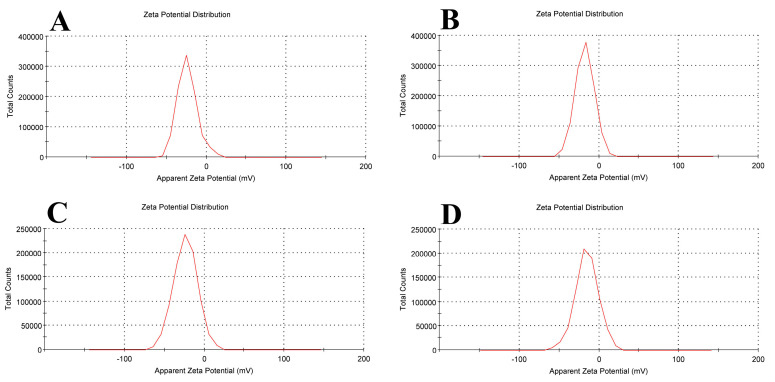
Zeta potential for (**A**) empty soy lecithin liposomal sample, (**B**) tamoxifen-encapsulated liposomes, (**C**) β-carotene-encapsulated liposomes, and (**D**) tamoxifen combined with beta-carotene into liposomes.

The liposomal formulation containing β-carotene and tamoxifen showed the lowest zeta potential (−15.2 ± 14.5 mV) because there was less particle repulsion, which resulted in a less stable colloidal dispersion (Figure 6). The results of zeta potential and dynamic light scattering measurements of various liposomal formulations are summarized in Table 1.

**Figure 7:**
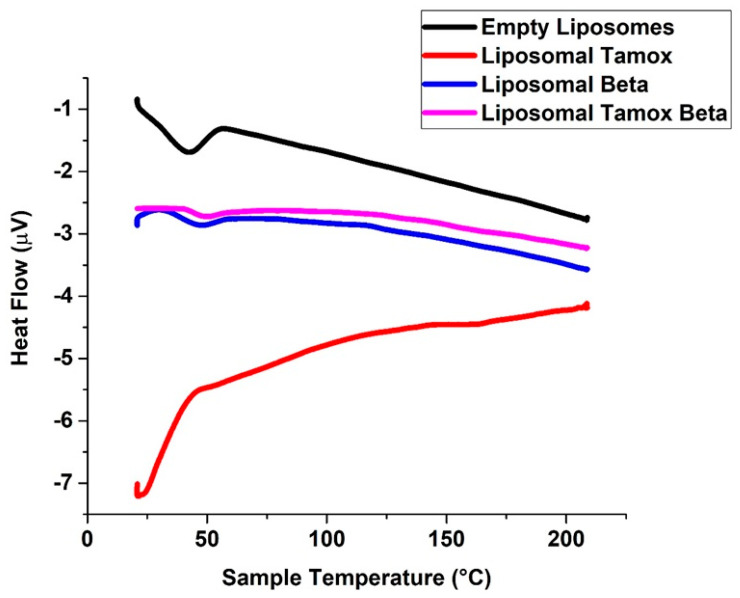
DSC diagrams of liposomes made of pure soy lecithin, liposomes doped with either tamoxifen, beta-carotene, or both.

Using DSC characterization, changes in the lipid bilayer’s phase transition temperature brought on by altered interactions between the liposomes and the encapsulated medications were examined [34,35]. The soy lecithin vesicles were used as model membranes because this phospholipid may resemble many properties of biological membranes. In agreement with Owusu-Ware et al. [36] and Shafaa et al. [37], pure soy lecithin vesicles showed a notable major endothermic peak (T_m_) at 41.6 °C after being dehydrated (Figure 7).

A substance in the soy lecithin membranes may have an impact on the vesicle transition’s thermo-tropic characteristics. When β-carotene or β-carotene combined with tamoxifen was incorporated into soy lecithin liposomes, the main endothermic peak (T_m_) of empty soy lecithin, which was present at 41.6 °C, significantly shifted to a higher temperature at 44 °C and 47 °C, respectively. This indicates that the β-carotene or β-carotene combined with tamoxifen had a significant effect on the acyl chains of soy lecithin bilayers, establishing a conformational order inside the phospholipids and increasing the cooperative transition of lipid acyl chains [38,39]. Figure 7 shows that the formation of tight and ordered acyl chains is more favorable when β-carotene or β-carotene combined with tamoxifen is incorporated. This is indicated by the increased temperature of vacant soy lecithin’s main endothermic peak (T_m_).

It is interesting to note that the main endothermic peak (T_m_) of empty soy lecithin, which presents at 41.6 °C, significantly shifted to a lower temperature at 21 °C when tamoxifen was added to soy lecithin liposomes. This suggests that tamoxifen had a significant effect on the soy lecithin bilayer of the acyl chains, causing phospholipid conformational disorder and decreasing the cooperative transition of lipid acyl chains. The main endothermic peak (T_m_) of empty soy lecithin showed a lower temperature, suggesting that the addition of tamoxifen promotes the formation of loose, disordered acyl chains.

As shown by DSC, the drug’s insertion between the polar heads of soy lecithin may promote the formation of a less organized liquid crystalline phase than the gel phase and marginally lower the gel-to-liquid crystal phase transition temperature [40]. Soy lecithin tamoxifen or β-carotene mixtures have been seen to exhibit a single peak using DSC, indicating that they are mixable [41]. FTIR, which has been used to assess any alteration in the liposomal membrane structure through wavenumber analysis of multiple vibrational modes, can verify some of the changes noticed in the current DSC work.

**Figure 8:**
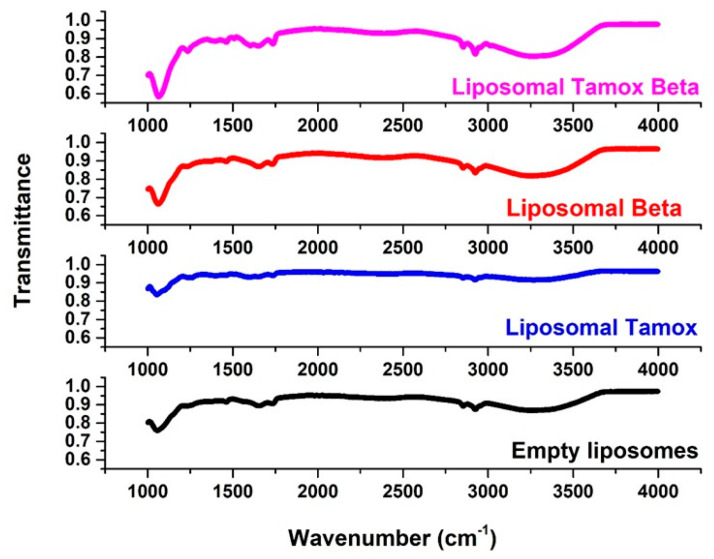
The full FTIR spectra of empty soy lecithin liposomes and liposomes doped with either tamoxifen, β-carotene, or both.

FTIR spectroscopy was used to examine the wavenumber of various functional groups, taking into account the acyl chains and the lipid molecule head group region in the presence or absence of foreign compounds, to analyze potential changes in the DSPC structure. The FTIR spectra of empty soy lecithin liposomes are displayed (Figure 8) and are compared with samples of soy lecithin liposomes and tamoxifen, β-carotene, or both in the 4000–400 cm^−1^ region.

The most elevated absorption FTIR distinctive peaks were seen in the soy lecithin liposome spectrum [42]. When β-carotene or tamoxifen are encapsulated into soy lecithin liposomes, the acyl chain’s symmetric CH_2_ stretching bands exhibit a different wavenumber (Figure 8). This suggests that the encapsulation of these compounds creates a conformational order in phospholipids’ acyl chains. Stated differently, they had an impact on the membrane’s order. When tamoxifen or β-carotene are doped with liposomes, the peak at 2853 cm^−1^ for pure soy lecithin is altered toward lower wave number 2852.61 cm^−1^ or 2852 cm^−1^, respectively. This might point to a reduction in gauche conformers, which would suggest a rise in bilayer order [43]. It is interesting to note that the tamoxifen-loaded liposomes’ signal intensity has decreased. When tamoxifen and β-carotene are added to soy lecithin liposomes, the peak of 2853 cm^−1^ for pure soy lecithin is moved to the higher wave number 2858 cm^−1^, indicating an increase in membrane fluidity and consequent destabilization of the system in the gel phase. When tamoxifen was added along with β-carotene (2920 cm^−1^) or β-carotene alone (2920 cm^−1^), the CH_2_ antisymmetric stretching band wavenumber changed toward a lower wavenumber compared to those of pure soy lecithin liposomes (2923 cm^−1^) (Figure 8).

One sensitive indicator of alkyl chain ordering in alkyl chains is the peaks of symmetric and asymmetric stretching vibrations of CH_2_. Significant alterations in the wavenumber of the CH_2_ stretching band indicate that β-carotene reduced the gauche conformer number, implying a rise in the order of bilayer conformation [44]. The interaction between β-carotene, tamoxifen, or tamoxifen combined with β-carotene and the glycerol backbone near the head group of phospholipids in the interfacial area is examined through the C=O stretching band [45]. As can be observed in (Figure 8), liposomal samples containing tamoxifen, β-carotene, or tamoxifen in combination with β-carotene have an increased wavenumber value of the C=O group at 1732 cm^−1^, without any sign of hydrogen bonding formation. Changes in the ester C=O stretching contours of the soy lecithin liposome molecule regulated the extent of hydrogen-bond creation in the glycerol backbone area.

Based on empirical rules, a decrease in frequency values indicates stronger hydrogen bonding or the formation of new hydrogen bonds between the components [43]. The C=O ester absorption bands are influenced by hydrogen bonding and other interactions, as well as variations in the polarity of their local surroundings. Thus, any change in this region’s spectrum could be the result of tamoxifen or β-carotene interacting with the membrane’s polar/apolar interfacial region [46].

The relationship between the head group of soy lecithin liposomes and tamoxifen, β-carotene, or a combination of both was investigated through the PO_2_^−^ antisymmetric stretching band (1241 cm^−1^). The PO_2_^−^ antisymmetric stretching band for soy lecithin liposome formulations with and without tamoxifen, β-carotene, or a combination of both is displayed in Figure 8. Furthermore, the wavenumber decreased upon the incorporation of β-carotene (1228 cm^−1^) into soy lecithin liposomes. This indicated the presence of hydrogen bonding between the liposome’s head group and β-carotene. When the wavenumber value decreases, it indicates that either new or strengthened hydrogen bonds are formed between the components [43].

Apolar carotenoid β-carotene is completely submerged and dispersed randomly, with no preferred well-defined orientation, in the bulk hydrophobic interior of the bilayer while maintaining a considerable degree of mobility. The lipid polar groups’ motional freedom at the lipid–water interface and the fatty chains’ liquid crystalline state are both improved by the presence of β-carotene in PC liposomes [28]. Xanthophylls, also known as polar carotenoids, are immobilized within the membrane while crossing the double lipid layer that anchors their hydroxyl groups in the polar region of the lipid headgroups. They are positioned nearly perpendicular to the membrane plane [47].

Conversely, nonpolar β-carotene is likely deeply enmeshed in the lipid bilayer and interacts with phospholipid molecules’ phosphate groups much less strongly. After being incorporated into soy lecithin liposomes, Table 2 displays the chemical shifts noticed for either β-carotene, tamoxifen, or a combination of both.

### 3.2. Cytotoxicity Effects of Tamoxifen Combined with β-Carotene into Liposomes Toward Noncancerous and Cancerous Cells

**Figure 9:**
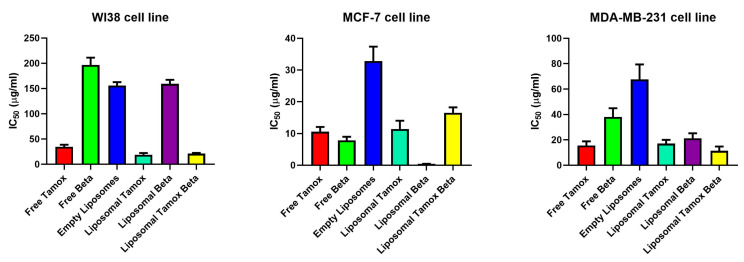
IC_50_ values for breast carcinoma cell lines (WI38, MCF-7, and MDA-MB-231) after 48 h exposure to various treatments: free tamoxifen, free β-carotene, empty liposomes, tamoxifen-loaded liposomes, β-carotene-loaded liposomes, and combination of tamoxifen/β-carotene-loaded liposomes. Determined by MTT assay.

The medication delivery system’s effectiveness was evaluated against human breast cancer cell lines MCF-7 and MDA-MB-231 utilizing the in vitro cytotoxicity (MTT) assay at various drug concentrations of free β-carotene, tamoxifen, or a combination of both and separately their liposomal forms [25]. Cells that had not been treated served as monitors at zero drug concentrations. Separately, the cancer cell line was incubated at various drug doses with the same sequence starting from 100 to 1200 µg/mL, for 48 h. After 48 h, the test was stopped, and cell viability was assessed (Figure 9).

Cell viability measurements showed that free β-carotene had the greatest rate of cytotoxicity on MCF-7 cell lines dealt with the same sequence of various concentrations provided to other medications. Forty-eight hours after incubation, MCF-7 treated cells showed a cell viability of roughly 10% at the maximum β-carotene concentration (1200 μg/mL). Cell viability for cells treated with β-carotene-loaded liposomes was roughly 20%, while for cells treated with tamoxifen-loaded liposomes, it was roughly 15% as opposed to 11% for free tamoxifen at the same concentration (1200 µg/mL).

The drug’s entrapment within several lipoidal domains of vesicles is the cause of its decreased efficacy in the lipo-solubilized state. Interestingly, when treated with the same concentration (1200 µg/mL), empty liposomes showed a significant decline in cell viability against MCF-7 cell lines (14% cell viability). This demonstrates that the high concentration of liposomes poisoned the tested cells. Different mechanisms may be responsible for the interferences with cell proliferation that we noticed in conjunction with certain phosphatidylcholines, as there is no clear rule regarding toxicity. These underlying mechanisms have only been the subject of conjecture thus far; future work will concentrate on a more thorough examination.

The development of colon cancer, the trigger of cell cycle arrest, and apoptosis were all inhibited by β-carotene. It has been verified that β-carotene is in charge of lowering cyclin A, a key factor in controlling the progression of the cell cycle [48]. The hypothesis that this carotenoid is either chemotherapeutic or chemopreventive for specific cancer types, such as prostate cancer [49] and melanoma [50], is supported by in vitro research. Interestingly, when tested against MCF-7 cell lines at the same concentration (1200 µg/mL), the combination of tamoxifen and β-carotene showed a significant reduction in cell viability. The viability of the cells was about 20%. At higher concentrations, β-carotene and tamoxifen appear to have the potential to work in synergism.

In the cytotoxic assay using MCF-7 treated cells, the IC_50_ value for liposomal β-carotene was found to be a minimum of 0.45 μg/mL, whereas the amount of free-β-carotene-treated cells was 7.8 μg/mL. For MCF-7 cells treated with free tamoxifen, the IC_50_ was 10.6 μg/mL, while for its liposomal form, it was 11.4 μg/mL (Figure 9). According to the cytotoxicity results using MDA-MB-231 treated cells, the IC_50_ values for free tamoxifen, free β-carotene, liposomal tamoxifen, liposomal β-carotene, and liposomal tamoxifen β-carotene were 15.5 μg/mL, 38.1 μg/mL, 17.1 μg/mL, 21.2 μg/mL, and 11.4 μg/mL, respectively. Those of the normal WI38 cell line were 34.7 μg/mL, 196.8 μg/mL, 18.9 μg/mL, 159.4 μg/mL, and 21.2 μg/mL, respectively, for free tamoxifen, free β-carotene, liposomal tamoxifen, liposomal β-carotene, and liposomal tamoxifen β-carotene. The reason for this enhanced effectiveness could be the drug’s lipo-solubilized state, similar to that of β-carotene, which is trapped in several lipoidal domains of vesicles. Furthermore, at the vesicle’s periphery, the phospholipid lamella can merge with the cell membrane to allow the vesicular contents to be internalized. Figure 9 shows that liposomal β-carotene has the highest therapeutic effectiveness against the MCF-7 cell line, relying on the type of cancer cells, based on the above results. The cytotoxic assay using MCF-7 reveals that the liposomal tamoxifen β-carotene IC_50_ was 16.66 μg/mL.

Pharmacokinetic and pharmacodynamic variables are among the reasons for the inconsistent toxicity of liposomal beta-carotene and liposomal tamoxifen. Variations include the enhanced cellular uptake of beta-carotene brought on by liposomes, which can raise intracellular concentrations and increase toxicity. This is linked to altered bioavailability, where beta-carotene’s bioavailability may be altered via liposomal encapsulation, increasing its cell accessibility [51]. Because beta-carotene is extremely lipophilic, which may improve its incorporation into liposomes and increase its toxicity [52], drug-specific variables might also result in inconsistent toxicity between liposomal beta-carotene and liposomal tamoxifen. Due to its lower lipophilicity, tamoxifen would not gain as much from liposomal formulation. The antioxidant qualities of beta-carotene at excessive doses can turn pro-oxidant, increasing toxicity [53]. The liposomal formulation may not enhance the estrogen-receptor-mediated action of tamoxifen. Last but not least, biological factors may contribute to this pattern of inconsistency in toxicity between liposomal beta-carotene and liposomal tamoxifen. This is because cells may metabolize liposomal beta-carotene differently than free beta-carotene, which increases toxicity and makes it possible for liposomal beta-carotene to trigger particular cell death pathways [51], while the mechanism of tamoxifen may not be as impacted by liposomal formulation. The improved intracellular drug accumulation by nanoparticle uptake may be the mechanism behind liposomal beta-carotene’s increased potency against MCF-7, according to the aforementioned arguments [54]. The benefit of liposomal beta-carotene over β-carotene free medicine, however, is that a single dose of liposomal beta-carotene will have a considerably longer pharmacological activity (sustained) than a single dose of free drug [51]. Additionally, because of its improved permeability and retention effect, liposomal beta-carotene may offer passive targeting [55]. The size of the nanoparticles may affect their cellular uptake by MCF-7 breast cancer cells; hence, the preferential uptake of the nanoparticles over the free medication may be the cause of the higher toxicity.

### 3.3. Tamoxifen Combined with β-Carotene into Liposomes Induced DNA Damage

**Figure 10:**
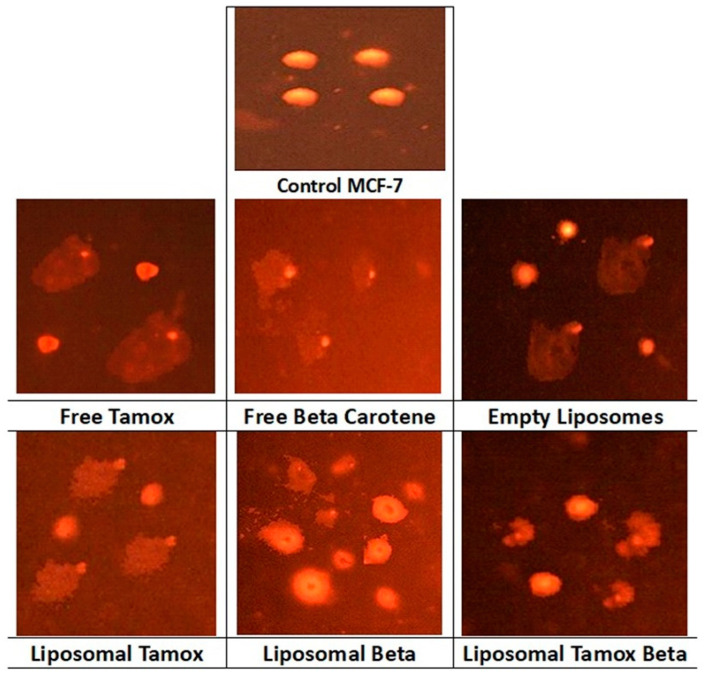
Comet assay images of MCF-7 cell line (evaluation of DNA damage induced by free tamoxifen, free β-carotene, empty liposomes, and liposomes doped with either tamoxifen, β-carotene, or tamoxifen combined with β-carotene compared to control MCF-7).

Figure 10 provides an analysis of the comet assay photographs for each formulation added to the MCF-7 cell line. The photo’s red circular spot indicates the intact DNA without migration, while the comet-shaped area next to the nucleus represents DNA breaks small enough to migrate in the gel and signifies the undamaged control cell. The DNA was packed closely together, and the typical circular orientation of the nucleus was preserved. The comet’s image for the nuclear DNA profile was changed by the presence of a streak of fluorescence that extended from the nucleus. Cells containing damaged DNA had the appearance of a comet with a bright tail and head.

**Figure 11:**
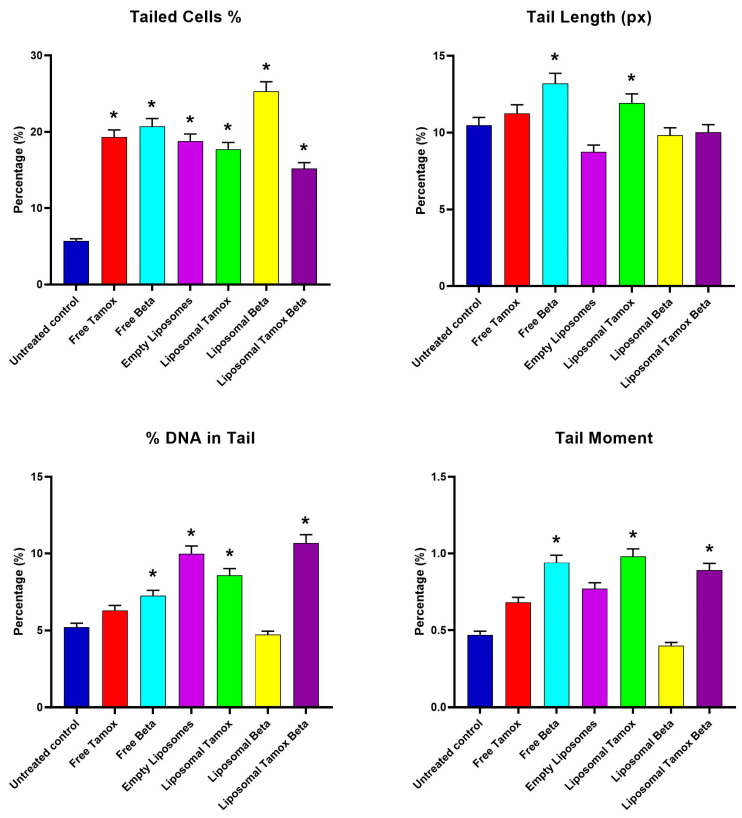
Comet assay parameters (tailed cells %, tail length, DNA in tail %, and tail moment) for control and treated groups. Data are reported as mean ± standard error from three independent experiments. Statistical analysis was performed by unpaired *t*-test to evaluate continuous variable differences between two groups. * *p*-values ≤ 0.05 were considered significant.

Figure 11 showed that the comet tail intensity is higher in MCF-7 cells treated with β-carotene-loaded liposomes than in cells treated with β-carotene alone. There are a considerable number of double-strand breaks because of the comet tail’s higher intensity than its head. MCF-7 cells treated with plain β-carotene showed a higher percentage of mortality. These findings support the theory that a nanoscale delivery system can more quickly and efficiently reach cancer cells than its macroscale equivalent. In contrast to control and liposomal tamoxifen-treated cells, MCF-7 cells treated with free tamoxifen show significantly more DNA damage (*p* < 0.05). The percentage of DNA in the tail provides a numerical representation of the amount of damaged DNA since the tail’s length and density indicate the quantity of single-strand breaks in the molecule. Another sign of DNA damage is an elevated mean tail moment.

For the MCF-7 control cell line, Figure 11 shows the comet assay variables (tail length, tail moment, percent of tail cells, and percentage of tail DNA) and post-treatment with empty liposomes, free tamoxifen, free β-carotene, and liposomes coupled with either tamoxifen, β-carotene, or tamoxifen combined with beta-carotene separately, as opposed to the control group, as well as the differences between the control and post-treatment groups. The findings demonstrated that all comet assay variables were considerably (*p* < 0.05) higher for the liposomal β-carotene group than for the control values.

Gloria et al. [56] reported that β-carotene selectively hinders the development of breast cancer. There is proof to suggest that either free β-carotene or its nanoliposomal form has a greater effect on killing breast cancer cells. Their findings provide new insight into a novel therapeutic regimen wherein cyclophosphamide could be replaced with liposomal β-carotene or β-carotene to increase the anticancer activity against the MCF-7 cancer cells.

### 3.4. Tamoxifen Combined with β-Carotene into Liposomes Stimulates Apoptosis in MCF-7 Cells

**Figure 12:**
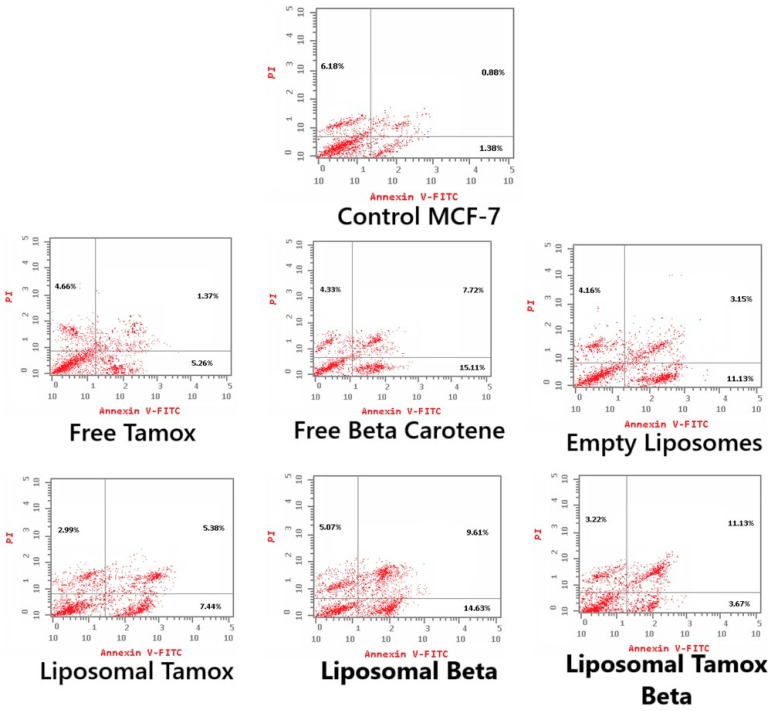
Representative flow cytometric analysis of MCF-7 cells: Control MCF-7, treated with the IC_50_ concentration of free tamoxifen, free β-carotene, empty liposomes, and liposomes doped with either tamoxifen, β-carotene, or tamoxifen combined with β-carotene for 48 h. The PI axes scale is reported as linear, and the Annexin V-FITC axes scale is reported as logarithmic.

**Figure 13:**
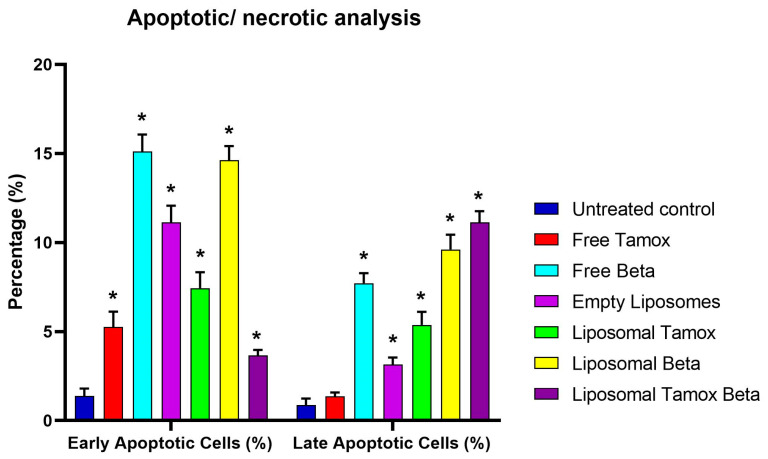
Results from MCF-7 cells: Control MCF-7, treated with the IC_50_ concentration of free tamoxifen, free β-carotene, empty liposomes, and liposomes doped with either tamoxifen, β-carotene, or tamoxifen combined with β-carotene for 48 h are expressed as a percentage of the total population. Data are reported as mean ± standard error from three independent experiments. Statistical analysis was performed by unpaired *t*-test to evaluate continuous variable differences between two groups. * *p*-values ≤ 0.05 were considered significant.

Using Annexin V-FITC/PI staining, Figure 12 and Figure 13 show the various stages of apoptosis induced in MCF-7 cells by empty liposomes, free tamoxifen, free β-carotene, and liposomes doped with either tamoxifen, β-carotene, or tamoxifen combined with β-carotene separately. For the IC_50_ concentration of all investigated samples, the graph displays the percent of cells in each phase of apoptosis, including viable cells, early apoptotic cells, late apoptotic cells, and necrotic cells. According to the findings, MCF-7 cells exposed to the IC_50_ concentration of liposomal β-carotene showed the highest percent of early and late apoptosis (29.31%), which was followed by free β-carotene (27.16%). On the contrary, when cells were subjected to the IC_50_ concentration of free tamoxifen, the lowest percent of both early and late apoptosis (11.29%) was seen. Furthermore, just a tiny percentage of the characteristic apoptotic features were present in untreated cells.

Our study found that administering liposomal β-carotene at an IC_50_ concentration to MCF-7 cancer cells resulted in a considerable boost in apoptosis when compared to free tamoxifen, implying a relationship between reduced liposomal β-carotene resistance and increased apoptosis. Measurements were made of the apoptotic marker Annexin V expression to assess the impact of liposomal β-carotene on cell viability. After 48 h of exposure to liposomal β-carotene, flow cytometric analysis showed that the MCF-7 cells had greater overall numbers of apoptotic cell populations (Q2 + Q4) than the control cells. This suggests that the MCF-7 cells were less resistant to the effects of liposomal β-carotene.

## 4. Conclusions

The available data provide insight into an innovative treatment regimen wherein liposomal β-carotene could be used in place of tamoxifen to increase the anticancer activity against the MCF-7 cancer cell line. There is verification that liposomal β-carotene has a greater effect on eradicating breast cancer cells. The use of liposomes as a medication delivery mechanism has an important role to play in reducing the rate of drug release, as predicted by the study of nanoliposomal drug release patterns, which indicate that drug release is fairly slow. Liposomal β-carotene, a natural treatment, may be the best alternative for treating human breast cancer MCF-7 cells without causing the many side effects associated with chemotherapy. This finding will allow for the reduction in cytotoxic agent dosages, leading to more targeted and less toxic medications for the management of human breast cancer. It also offers a molecular foundation for the synthesis of new anticancer compounds derived from natural sources. Future research is advised to investigate different natural liposome combinations with alternative cancer treatments as well as decipher their mode of action and potential effectiveness in vivo. The current findings demonstrated that natural product preparations might be a suitable substitute for pharmaceutical interventions in the treatment of breast cancer.

## Figures and Tables

**Table 1:** Summarized data obtained for the dynamic light scattering (DLS) and zeta potential for liposomes before and after encapsulation by beta-carotene, tamoxifen, or tamoxifen combined with beta-carotene.

Sample Name	Mean Size Diameter (nm) ± SD (nm)	PDI Average	Mean Zeta Potential ± SD (mV)
Empty Liposomes	43.82 ± 21.86	0.477	−23.8 ± 12.4
Liposomal Tamox	91.28 ± 22.13	0.546	−17.6 ± 11.7
Liposomal β-carotene	91.28 ± 27.26	0.674	−23.5 ± 14.9
Liposomal Tamox + Beta	122.4 ± 83.37	0.408	−15.2 ± 14.5

**Table 2:** The chemical shifts observed for tamoxifen, β-carotene, and a mixture of them after the incorporation into soy lecithin liposomes.

Peak Assignment	Wavenumber (cm^−1^)	Wavenumber (cm^−1^)			
		Control	Tamoxifen	Beta-Carotene	Tamox Mixed with Beta
Symmetric stretching vibration of CH_2_ in acyl chain	(2800–2855)	2853	2852.61	2852	2858
Antisymmetric stretching vibration of CH_2_ in acyl chain	(2916–2921)	2923	2926. 61	2920	2920
Carbonyl stretching vibration C=O	(1730–1740)	1732	1735.58	1741	1735
Antisymmetric PO_2_^−^ stretching vibrations	(1215–1260)	1241	1241	1228	1241

## Data Availability

We confirm that all original raw data are available at the time of submission. As per the Data Policy, these data will be stored for a minimum of 10 years and will be made available to the Editorial Office, Editors, and readers upon request.
